# Eucaine: A New Local Anæsthetic

**Published:** 1898-07

**Authors:** 


EUCAINE: A NEW LOCAL ANÆSTHETIC. Dentist Kiesel, of Berlin, writes to the *Drogisten Zeitung,* in regard to eucaine, that it has many advantages over cocaine, especially as the heart is in no way affected by it, the pulse remaining normal. Anæsthesia is more extended and lasts longer than in cocaine. Without injury to the health of the patient, 2 gr. of eucaine can be injected to 0.01 gr. of cocaine per dose. From a solution 1 : 6½—which would correspond to a 15 per cent. solution—twelve syringefuls could be injected before reaching the maximum dose. But usually half this quantity is sufficient to render the extraction of all the teeth painless. Solutions of 1 : 6½, made with sterilized water, remain clear in ordinary temperatures, and never become flaky without the addition of carbol or salicyl, as cocaine does. We may then conclude that the ease of application, the certainty of the result, and, most of all, the great advantage over other local anæsthetics, that we have no unpleasant after-effects, will soon make eucaine a very popular anæsthetic.—*Idem.*

